# Polyoxometalate chemistry at volcanoes: discovery of a novel class of polyoxocuprate nanoclusters in fumarolic minerals

**DOI:** 10.1038/s41598-020-63109-1

**Published:** 2020-04-14

**Authors:** S. N. Britvin, I. V. Pekov, V. O. Yapaskurt, N. N. Koshlyakova, J. Göttlicher, S. V. Krivovichev, A. G. Turchkova, E. G. Sidorov

**Affiliations:** 1 Department of Crystallography, Institute of Earth Sciences, St. Petersburg State University, University Embankment 7/9, 199034 St Petersburg, Russia; 2 Kola Science Center of Russian Academy of Sciences, Fersman Str. 14, 184200 Apatity, Russia; 3 Faculty of Geology, Moscow State University, Vorobievy Gory, 119991 Moscow, Russia; 4 Karlsruhe Institute of Technology, Institute for Synchrotron Radiation, Hermann-von-Helmholtz-Platz 1, D-, 76344 Eggenstein-Leopoldshafen, Germany; 5 Institute of Volcanology and Seismology, Far Eastern Branch of the Russian Academy of Sciences, Piip Boulevard 9, 683006 Petropavlovsk-Kamchatsky, Russia

**Keywords:** Solid-state chemistry, Geochemistry, Mineralogy

## Abstract

Polyoxometalate (POM) chemistry is an important avenue of comprehensive chemical research, due to the broad chemical, topological and structural variations of multinuclear polyoxoanions that result in advanced functionality of their derivatives. The majority of compounds in the polyoxometalate kingdom are synthesized under laboratory conditions. However, Nature has its own labs with the conditions often unconceivable to the mankind. The striking example of such a unique environment is volcanic fumaroles – the natural factories of gas-transport synthesis. We herein report on the discovery of a novel class of complex polyoxocuprates grown in the hot active fumaroles of the Tolbachik volcano at the Kamchatka Peninsula, Russia. The cuboctahedral nanoclusters {[*M*Cu_12_O_8_](AsO_4_)_8_} are stabilized by the core Fe(III) or Ti(IV) cations residing in the unique cubic coordination. The nanoclusters are uniformly dispersed over the anion- and cation-deficient NaCl matrix. Our discovery might have promising implications for synthetic chemistry, indicating the possibility of preparation of complex polyoxocuprates by chemical vapor transport (CVT) techniques that emulate formation of minerals in high-temperature volcanic fumaroles.

## Introduction

Polyoxometalates (POMs) constitute a large group of materials with discrete metal-anion clusters of various shapes and sizes^1–3^. Traditionally, POMs were associated with *d*-block metals in high oxidation states (V^5+^, Nb^5+^, Ta^5+^, Mo^6+^ and W^6+^), but recent studies extended the field to other elements such as actinides^4^ and noble metals^5^. In 1990, Achim Müller and co-workers^6^ introduced the term ‘polyoxocuprates’ (POCus) to identify clusters formed by polymerization of Cu coordination polyhedra, previously reported for synthetic inorganic compounds such as Ba_44_Cu_45_O_87_Cl_4_ and Ba_88_Cu_88_O_175_Br_2_^7^. The POCus have been intensively investigated recently as reviewed by Kondinski and Monakhov^8^, due to their potential applications in catalysis, molecular magnetism and superconductivity. Usually POCus are obtained by crystallization from aqueous solutions at pH values between 3.5 and 7 *via* polymerization of Cu(OH)_4_ planar-square units.

Natural crystalline POMs have been under extensive investigation over the last ten years and are known for Mo^9^, V^10–12^, and Nb^13^. Besides, uranyl carbonate nanoscale clusters have been reported for U^6+^ carbonate minerals such as ewingite, Ca_8_Mg_8_(UO_2_)_24_(CO_3_)_30_O_4_(OH)_12_(H_2_O)_138_^14^, and paddlewheelite, Ca_5_MgCu_2_(UO_2_)_4_(CO_3_)_12_(H_2_O)_33_^15^. Other examples are two natural arsenates, bouazzerite^16^, Bi_6_(Mg,Co)_11_Fe_14_(AsO_4_)_18_O_12_(OH)_4_(H_2_O)_86_, and whitecapsite^17^, H_16_Sb^3+^_6_Fe^2+^_5_Fe^3+^_14_(AsO_4_)_18_O_16_(H_2_O)_120_, which are based upon heptanuclear iron-oxide-arsenate nanoclusters. We note that discrete Cu-OH clusters have also been reported in minerals, *e.g*., the [Cu_12_(OH)_24_] clusters in zeolite tschörtnerite^18^, Ca_4_(Ca,Sr,K,Ba)_3_Cu_3_[Al_3_Si_3_O_12_]_4_(OH)_8_·*n*H_2_O, the [Cu_24_(OH)_48_] clusters in boleite^19^, KPb_26_Ag_9_Cu_24_(OH)_48_Cl_62_, and pseudoboleite^20^, Pb_31_Cu_24_Cl_62_(OH)_48_, and the [Cu_20_(OH)_40_] clusters in cumengeite^21^, Pb_21_Cu_20_Cl_42_(OH)_40_·6H_2_O. It is noteworthy that all the reported occurrences of POMs in nature have been restricted to low-temperature aquiferous systems, namely oxidation zones of ore deposits (<50–70 °C) or last-stage hydrothermal environments (<150–200 °C).

Volcanic fumaroles are unique in both physical and chemical (geochemical) aspects. In fumarolic systems minerals either directly precipitate from volcanic gases as sublimates or form as a result of their interactions with host rocks. Among fumarolic minerals, copper-based anhydrous mineral phases constitute one of the most rich and diverse groups. The explosion of recent discoveries of Cu^2+^ fumarolic minerals is related to the Tolbachik volcano at Kamchatka Peninsula, Russia. The Cu mineralization at Tolbachik was reviewed by Pekov *et al*.^22^, and the most recent descriptions of new mineral species can be found in refs. ^23–26^. The Cu minerals found in Tolbachik fumaroles are remarkable by their structural architecture, which frequently contain polymeric Cu-oxo units based upon oxocentered OCu_4_ tetrahedra. These tetrahedra may polymerize to form structural units of different dimensionalities^27,28^. Herein we report two novel halide-arsenate mineral species, arsmirandite and lehmannite, found at Tolbachik that represent the first example of natural POCus and provide a unique insight into the ability of Cu^2+^ cations to form discrete nanoclusters in anhydrous natural environments. Our discovery also provide some important clues for the synthesis and chemical tuning of cubic POCus for further studies of their physical and chemical properties.

## Results

**Figure 1:**
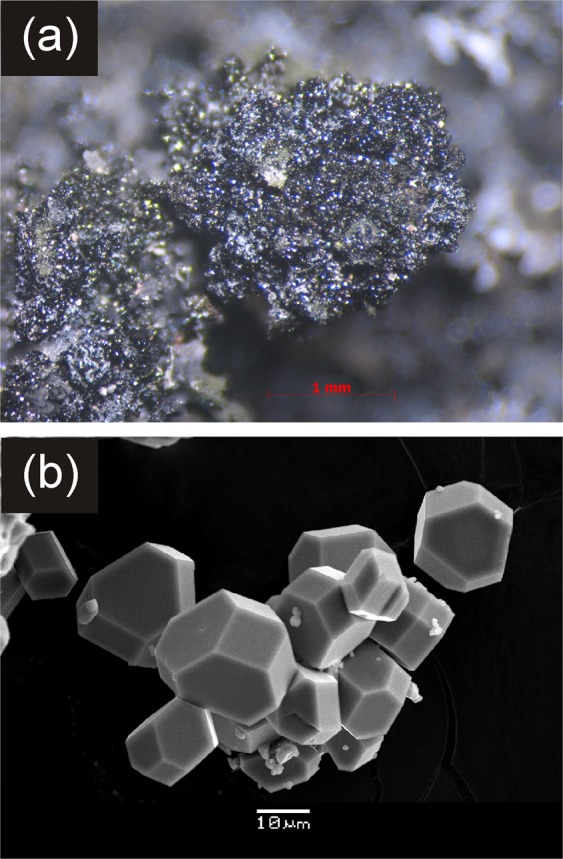
Crystalline crusts of greenish black arsmirandite (**a**) and scanning electron microscopy (SEM) image of clusters of arsmirandite crystals (**b**).

Two new mineral species, arsmirandite and lehmannite, have been found in the active Arsenatnaya fumarole^29,30^ located at the Second scoria cone of the Northern Breakthrough of the Great Tolbachik Fissure Eruption 1975–1976, Tolbachik volcano, Kamchatka Peninsula, Far-Eastern Region, Russia (55°41′N 160°14′E, 1200 m asl). The mineral assemblages encasing these halide-arsenates were formed in the temperature range between 500 and 700 °C^22,30^ and were located inside the fumarole. The detailed mineralogical description of the minerals will be given elsewhere. Polyoxocuprates occur as aggregates of greyish-green well-shaped crystals resembling rhombic dodecahedra (Fig. 1) that could easily be separated from the surrounding matrix. The chemical composition of arsmirandite and lehmannite were determined using electron-microprobe analysis (Table S0). The empirical formula of arsmirandite, calculated on the basis of 45 anions (O + Cl) *pfu*, is: Na_17.06_K_0.51_Ca_0.06_Pb_0.08_Mg_0.11_Mn_0.01_Cu_11.73_Zn_0.08_Al_0.02_ Fe^3+^_0.92_Ti_0.10_(As_7.91_S_0.08_P_0.03_Si_0.02_V_0.01_)_Σ8.05_O_40.23_Cl_4.77_. The empirical formula of lehmannite, calculated on the basis of the sum of tetrahedrally coordinated components (As+P + S + Si) = 8 *apfu*, is: Na_17.92_K_0.18_Ca_0.24_Cu_11.59_Fe^3+^_0.21_Ti_0.85_Sn_0.11_(As_7.74_S_0.14_P_0.09_Si_0.03_)_Σ8_O_40.10_F_0.75_Cl_5.42_. The idealized chemical formulae for arsmirandite and lehmannite were determined as Na_18_Cu_12_^2+^Fe^3+^O_8_(AsO_4_)_8_Cl_5_ and Na_18_Cu_12_^2+^Ti^4+^O_8_(AsO_4_)_8_FCl_5_, respectively.

**Figure 2:**
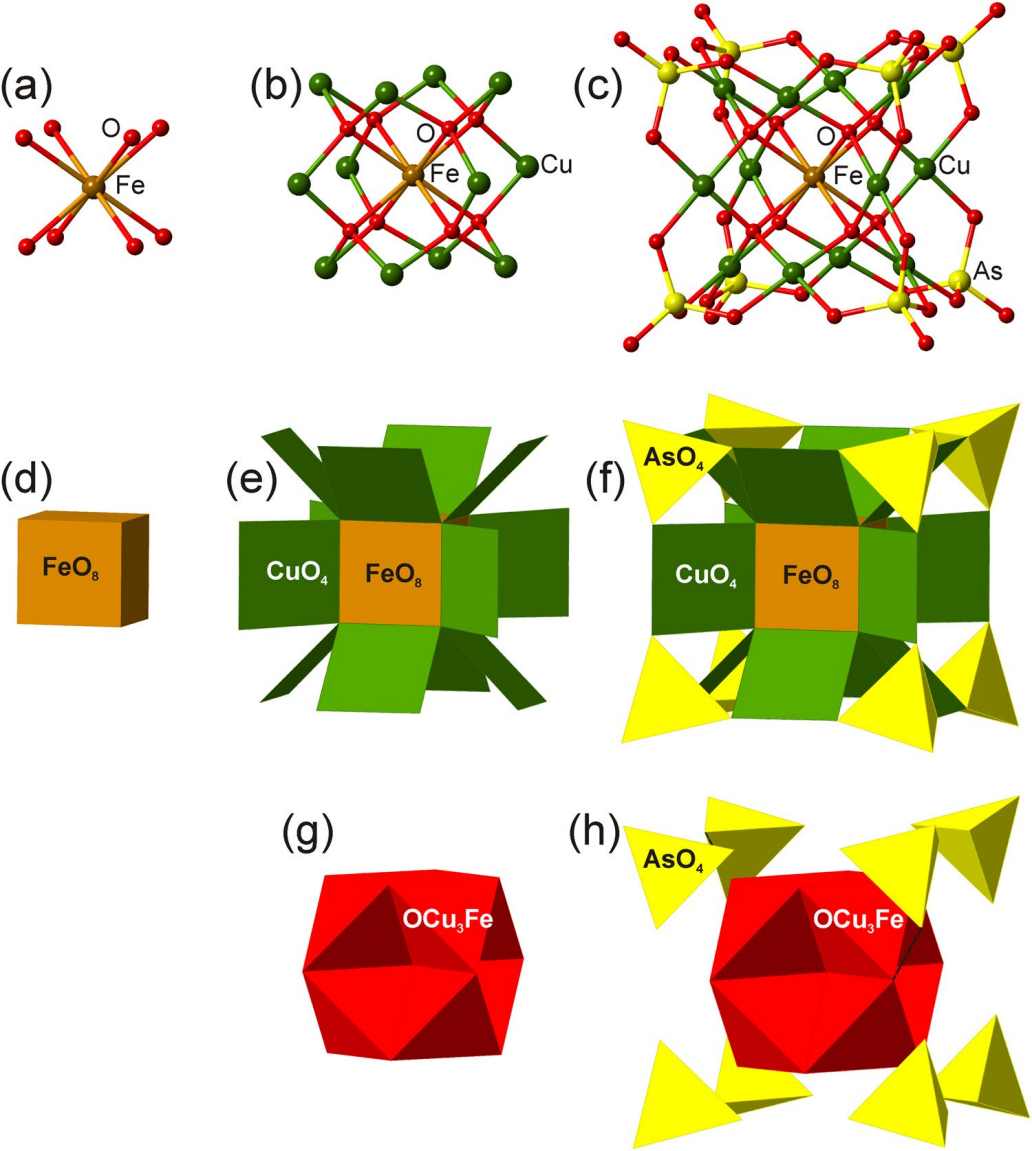
The structure of the polyoxocuprate nanoclusters in arsmirandite and lehmannite shown in ball-and-stick (**a–c**) and polyhedral (cation-centered (**d–f**) and combined anion- and cation-centered (**g**,**h**)) representations (exemplified by arsmirandite).

**Figure 3:**
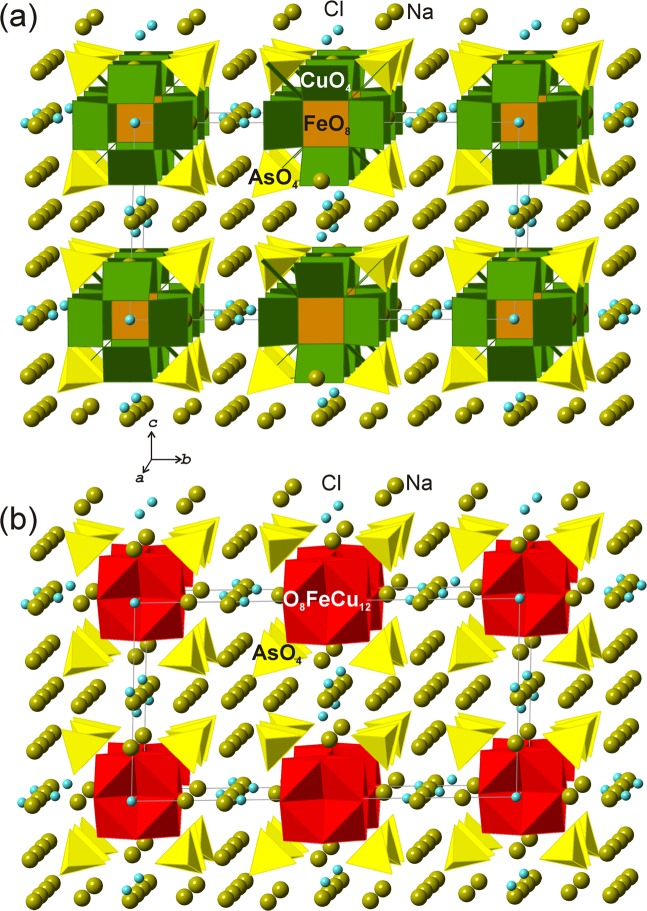
The crystal structure of arsmirandite with nanoclusters shown in cation-centered (**a**) and combined anion- and cation-centered (**b**) representations.

The crystal structures of arsmirandite and lehmannite (Tables S1–S7) are very similar, yet not identical. From the structural point of view, they are quite unusual and unique. The basic structural unit in both structures is a novel nanoscale (~1.5 nm across) polyoxocuprate cluster with the composition {[*M*Cu_12_O_8_](AsO_4_)_8_} (*M* = Fe^3+^ and Ti^4+^, for arsmirandite and lehmannite, respectively) shown in Fig. 2. The most peculiar feature of the nanocluster is the presence of Fe^3+^ (arsmirandite) or Ti^4+^ (lehmannite; the tetravalent state of Ti confirmed by XANES spectroscopy (Fig. S3)) in a cubic coordination (Fig. 2a): the central unit of the nanocluster represents the slightly distorted (*M*O_8_) cube (Fig. 2a,d). The cubic coordination of Fe(III) and Ti(IV) has not been encountered so far in natural minerals, though several examples are known for synthetic compounds^31–34^. Each O atom of the (*M*O_8_) configuration is further coordinated by three Cu^2+^ cations (Fig. 2b) that have square-planar geometry by O atoms of the (AsO_4_) groups (Fig. 2c,e,f). The metal-oxide core of the nanocluster can also be represented in terms of oxocentered (OCu_3_*M*) tetrahedra^27,28^ that form an eightfold unit (Fig. 2g), which can be considered as a fragment of the crystal structure of fluorite, if the latter is described as a framework of (FCa_4_) tetrahedra. The [O_8_*M*Cu_12_] core formed by eight oxocentered tetrahedra is surrounded by eight AsO_4_ tetrahedra that are in the face-to-face orientation relative to the (OCu_3_*M*) tetrahedra^35^ (Fig. 2h). The cubic nanoclusters are negatively charged and are surrounded by an array of Na^+^ cations and *X*^−^ anions (*X* = F, Cl) (Fig. 3), which requires further remark. The analysis of the Na array in arsmirandite and lehmannite (Figs. S1 and S2) shows that it is in fact a highly deficient face-centered cubic (*fcc*) lattice as observed, e.g. in the crystal structure of halite, NaCl^36^. The halite-like cubic pseudo-subcell can be obtained from the true unit cell using the matrix $(\frac{1}{2}00/0\frac{1}{4}0/\frac{1}{4}0\frac{1}{2})$, i.e. the structures of arsmirandite and lehmannite can be considered as the 2 × 4 × 4 supercell relative to the halite cell. The parameters of the halite-like cubic subcell, e.g., in lehmannite, are: *a*_hal_ = 5.418, *b*_hal_ = 5.273, *c*_hal_ = 5.279 Å, which shows that, realtive to halite, the deficient Na *fcc* array is compressed and tetragonally distorted.

## Discussion

The difference between arsmirandite and lehmannite is in the nature of the *M* cation, which triggers the re-arrangement of the halogen substructure. Since Fe^3+^ and Ti^4+^ possess different charges, the Fe^3+^-for-Ti^4+^ substitution requires the mechanism of charge compensation, which is achieved *via* the incorporation of the additional anionic site, F, in lehmannite (Ti^4+^ species) relative to arsmirandite (mineral with Fe^3+^). The charge compensation, therefore, is governed by the following substitution scheme:

$${{\rm{F}}{\rm{e}}}^{3+}+{\square }^{0}\to {{\rm{T}}{\rm{i}}}^{4+}+{{\rm{F}}}^{-},$$

where the ‘□’ sign stays for vacancy.

In lehmannite, the substitution is realized through the following crystal chemical mechanism. The Cl1 site with the 2/*m* site symmetry with the coordinates (1/2,1/2,0) in arsmirandite is shifted along the *a* axis to the Cl1 site with the *m* site symmetry (approximate coordinates: 0.53,1/2,0) in lehmannite, resulting in the splitting of the Cl1 site into two mutually exclusive sites. The F site with the same *m* site symmetry is incorporated (approximate coordinates: 0.65,1/2,0) into the structure so that the Cl1^…^F distance is equal to *ca*. 1.3 Å. The short distance precludes the simultaneous occupancy of the Cl1 and F sites with the maximum total occupancy F_0.50_Cl_0.50_. Note that the Cl1^…^Cl1 distance in disordered configuration is very short (~0.6 Å in lehmannite), prohibiting more-than-50% occupancy of the Cl1 site, whereas the F^…^F distance (~3.1 Å) allows for the full occupancy of the two F sites assuming that the Cl1 site is empty.

In lehmannite, the disordered configuration has the total occupancy F_0.52_Cl_0.34□0.16_ or F_1.04_Cl_0.68□0.32_ per formula unit (*pfu*) with the −1.72 total negative charge. The corresponding occupancy of the *M* site is calculated as Ti^4+^_0.69_Fe^3+^_0.28_Sn^4+^_0.03_ with the total positive charge +3.72, which provides the formula electroneutrality. The full substitution of Fe^3+^ by Ti^4+^ in lehmannite would correspond to the total occupancy of the anionic sites F_0.50_Cl_0.50_ or F_1.00_Cl_1.00_ *pfu* with the −2 total negative charge.

The crystal chemical formula of arsmirandite determined on the basis of chemical analysis and crystal-structure refinement can be written as (Na_17.54_K_0.46_)_Σ18.00_(Fe^3+^_0.78_Mg_0.11_Ti_0.11_)_Σ1.00_Cu^2+^_12_(AsO_4_)_8_O_8_Cl_5_. Note that the incorporation of Ti^4+^ into the *M* site is compensated by the coupled substitution of Fe^3+^ by Mg^2+^:

$$2{{\rm{F}}{\rm{e}}}^{3+}\to {{\rm{T}}{\rm{i}}}^{4+}+{{\rm{M}}{\rm{g}}}^{2+}.$$

The crystal chemical formula of lehmannite can be written as (Na_17.62_K_0.38_)_Σ18.00_(Ti^4+^_0.69_Fe^3+^_0.28_Sn^4+^_0.03_)_Σ1.00_Cu^2+^_12_(AsO_4_)_8_O_8_Cl_4.68_F_1.04_.

Taking into account the discussion given above, the general formula of the hypothetical arsmirandite-lehmannite series can be written as Na_18_(Ti^4+^_1-*x*_Fe^3+^_*x*_)Cu_12_ (AsO_4_)_8_O_8_Cl_6-*x-y*_F_*y*_. The ideal formula of arsmirandite corresponds to *x* = 1 and *y* = 0, whereas that of lehmannite requires *x* = 0 and *y* = 1. Note that 0 ≤ *x* ≤ 1, whereas 0 ≤ *y* ≤ 2. The experimental case of lehmannite corresponds to *x* = 0.28 and *y* = 1.04. The case *y* = 2 corresponds to the full occupancy of the F site and the complete emptiness of the Cl1 site and results in the ‘theoretical’ formula Na_18_(Ti^4+^_1-*x*_Fe^3+^_*x*_)Cu_12_(AsO_4_)_8_O_8_Cl_4-*x*_F_2_. For this formula, *x* = 1 results in the formula Na_18_Fe^3+^Cu_12_(AsO_4_)_8_O_8_Cl_3_F_2_ (hypothetical F-rich analogue of arsmirandite), whereas *x* = 0 corresponds to Na_18_Ti^4+^Cu_12_(AsO_4_)_8_O_8_Cl_4_F_2_ (hypothetical F-rich analogue of lehmannite). These two potentially possible species may occur in F-enriched fumarolic environments.

The metal-oxide [O_8_*Me*_13_] nanoclusters (*Me* = metal) consisting of eight (O*Me*_4_) tetrahedra sharing central *Me* atom are well-known in synthetic inorganic chemistry. It seems that the first detailed structural report was done for *Me* = Pb^2+^ found in the crystal structure of Pb_13_O_8_(OH)_6_(NO_3_)_4_^37^. In this compound, the central 8-coordinated cation in the nanocluster is Pb^2+^, which has a stereochemically inactive lone-electron pair, in contrast to twelve peripheral Pb^2+^ that possess strongly asymmetrical coordination environments. Later Kolitsch and Tillmanns^38^ reported Pb_13_O_8_(OH)_6_(NO_3_)_4_ as an anthropogenic compound formed in old mine dumps due to the use of nitrate explosives. The phase Pb_13_O_8_(OH)_6_(NO_3_)_4_ forms in the Pb(NO_3_)_2_-NaOH system at pH = 9-10^39^.

In 2008, Chubarova *et al*.^40^ reported on the synthesis and structure of Na_8_{[Pd_13_O_8_](AsO_3_(OH))_6_(AsO_4_)_2_}·42H_2_O, the compound, which opened up the whole new field of polyoxopalladate chemistry^5^. The crystal structure of this compound is based upon {[Pd_13_O_8_](AsO_3_(OH))_6_(AsO_4_)_2_}^8−^ nanoclusters structurally identical to those found in arsmirandite and lehmannite (which are also arsenates). Later it was found that arsenate groups can be replaced by phosphate^41^ or selenite groups^42–44^. The central Pd^2+^ cation in the Pd-based 13-nuclear nanoclusters (Fe^3+^ and Ti^4+^ in arsmirandite and lehmannite, respectively, play the same role as Pd^2+^ in this compound) can be replaced by Na^+^ ^45^, *REE*^3+^ (*REE* = rare-earth element^42,46^), divalent or trivalent metal cations *M*^2+^ (*M* = Sc^3+^, Mn^2+^, Fe^3+^, Co^2+^, Ni^2+^, Cu^2+^, or Zn^2+^), and tetravalent Sn^4+^ and Pb^4+^ cations^47^. Very recently, Bhattacharya *et al*.^48^ reported on the use of the 13-nuclear Pd-based nanoclusters, [Pd_13_O_8_(AsO_4_)_8_H_6_]^8−^, incrustated by Ba^2+^ cations for the construction of novel types of porous metal-organic frameworks. It is remarkable that, in arsmirandite and lehmannite, the role of Pd^2+^ cations in peripheral metal positions is played by Cu^2+^ cations, whereas the central cation in the metal-oxide core is either Fe^3+^ or Ti^4+^, respectively. The observed stereochemical similarity is due to the similar square planar coordination by O^2−^ anions specific for both Pd^2+^ and Cu^2+^. Our findings indicate the chemical possibility of the family of novel polyoxocuprate clusters with interesting functional properties. Yang and Kortz^5^ indicate that the Pd nanoclusters are stable in the solid state, solutions (both aqueous and organic) and gases, which allows for their applications in catalysis, nanotechnology, molecular spin qubits and in biology as aqueous-phase macromolecular models.

Kondinski and Monakhov^8^ identified targeted synthesis of the hypothetical [Cu⊂Cu_12_O_8_L_8_]^*q*−^ polyoxoanion (L = inorganic or organic ligand, e.g. arsenate, phosphate, selenite, *etc*.) with two different coordination modes of central and peripheral Cu^2+^ ions as an interesting challenge for synthetic chemistry of POCus. The discovery of the [*M*⊂Cu_12_O_8_(AsO_4_)_8_]^*q*−^ clusters (*M* = Fe^3+^, Ti^4+^) in arsmirandite and lehmannite found in Tolbachik fumaroles provides a useful clue for the targeted synthesis of their analogues under laboratory conditions. As it has been demonstrated previously^49–51^, fumarolic minerals with oxocentered cores can be conveniently synthesized using chemical vapor transport techniques. The occurrence of the [*M*⊂Cu_12_O_8_(AsO_4_)_8_]^*q*−^ nanoclusters in Tolbachik fumaroles testifies that they are stable under high temperatures (500–700 °C) at least in the gaseous and crystalline phases and may as well possess interesting physical and chemical properties. They could also play the role of metal transport forms in fumarolic gases, in agreement with the previous proposal^52^ about the similar geochemical role of tetranuclear (OCu_4_) clusters.

The geometrical similarity of the Na array in arsmirandite and lehmannite to that observed in halite, NaCl, allows to describe both minerals as consisting of cubic-shape nanoclusters periodically integrated into deficient NaCl matrix, a feature that is quite uncommon for inorganic materials. There has been a recent interest in salt-inclusion compounds (SICs), which possess hierarchical structures consisting of porous metal-oxide frameworks with voids filled with simple ionic salts^53^. The examples of natural SICs are averievite, Cu_5_O_2_(VO_4_)·*nM*Cl_*x*_ (*M* = Cu, Cs, Rb, K)^54–57^, and aleutite, Cu_5_O_2_(AsO_4_)(VO_4_)·(Cu_0.5_□_0.5_)Cl^25^. In the case of arsmirandite and lehmannite, we have the opposite situation, *i.e*., the incorporation of metal-oxide clusters into the salt matrix, which, as to our knowledge, had never been observed at least in minerals and mineral phases.

## Supplementary information

Supplementary Information.
