# Supplementary material for: Polyoxometalate chemistry at volcanoes: discovery of a novel class of polyoxocuprate nanoclusters in fumarolic minerals
Source: Sci Rep. 2020 Apr 14;10:6345. doi: 10.1038/s41598-020-63109-1 (PMC7156706; doi:10.1038/s41598-020-63109-1)
Supplement: Supplementary file 1 — Supplementary Information. [file 41598_2020_63109_MOESM1_ESM.pdf]

SUPPLEMENTARY INFORMATION

Polyoxometalate chemistry at volcanoes: discovery of a novel class of polyoxocuprate nanoclusters in fumarolic minerals

S.N. Britvin, I.V. Pekov, V.O. Yapaskurt, N.N. Koshlyakova, J. Göttlicher, S.V. Krivovichev, A.G. Turchkova & E.G. Sidorov

Chemical data for arsmirandite and lehmannite were obtained using a Jeol JSM-6480LV scanning electron microscope equipped with an INCA-Wave 500 wavelength-dispersive spectrometer (Laboratory of Analytical Techniques of High Spatial Resolution, Dept. of Petrology, Moscow State University).

Table S0. Chemical composition (wt.%) of arsmirandite and lehmannite.

Constituent	Arsmirandite			Lehmannite			Probe standard
	Mean (5 anal.)	Range	SD	Mean (8 anal.)	Range	SD	
Na_2O	20.04	19.53 – 20.61	0.43	20.62	19.87 – 22.91	1.03	lorenzenite
K_2O	0.91	0.79 – 1.05	0.10	0.31	0.25 – 0.36	0.03	orthoclase
CaO	0.12	0.09 – 0.16	0.03	0.51	0.09 – 0.72	0.18	$CaWO_4$
PbO	0.67	0.59 – 0.72	0.05	-			PbTe
MgO	0.17	0.06 – 0.24	0.07	-			diopside
MnO	0.03	0.00 – 0.06	0.02	-			Mn
CuO	35.37	34.77 – 35.89	0.46	34.24	32.75 – 35.14	0.76	$CuFeS_2$
ZnO	0.25	0.18 – 0.36	0.07	-			ZnS
Al_2O_3	0.03	0.00 – 0.06	0.02	-			Al_2O_3
Fe_2O_3	2.79	2.38 – 3.07	0.27	0.63	0.37 – 0.88	0.20	$CuFeS_2$
TiO_2	0.29	0.19 – 0.40	0.08	2.53	1.96 – 3.70	0.53	ilmenite
SnO_2	-			0.62	0.12 – 1.37	0.38	SnS
SiO_2	0.05	0.00 – 0.10	0.04	0.06	0.02 – 0.10	0.03	diopside
P_2O_5	0.07	0.05 – 0.11	0.02	0.23	0.13 – 0.32	0.06	GaP
V_2O_5	0.04	0.00 – 0.09	0.03	-			V
As_2O_5	34.46	34.20 – 34.75	0.24	33.04	31.84 – 34.28	0.72	FeAsS
SO_3	0.25	0.17 – 0.37	0.09	0.43	0.00 – 2.67	0.43	ZnS
F	-			0.53	0.48 – 0.60	0.05	MgF_2
Cl	6.41	6.21 – 6.49	0.11	7.13	6.94 – 7.58	0.19	NaCl
–O=(F,Cl)	–1.45			–1.83			
Total	100.50			99.05			

SD – standard deviation; dash means that the content of a constituent is below detection limit.

Single-crystal X-ray studies of both new minerals were carried out using a Bruker Smart Kappa Apex DUO diffractometer equipped with an APEXII CCD detector. The crystal structures of arsmirandite and lehmannite were solved by direct methods and refined with the use of *SHELX*-2014 software package[1] to R = 0.028 and 0.046, respectively. Crystal data, data collection and structure refinement details for both minerals are presented in Tables S1 and S2, atom coordinates and displacement parameters are given in Tables S3–S6. Comparison of interatomic bond lengths for arsmirandite and lehmannite is presented in Table S7.

Table S1. Crystal data, data collection and structure refinement details for arsmirandite.

**Crystal Data**	
Formula	$(Na_{17.54}K_{0.46})_{\Sigma 18.00}(Fe^{3+}_{0.78}Mg_{0.11}Ti_{0.11})_{\Sigma 1.00}Cu^{2+}_{12}(AsO_4)_8O_8Cl_5$
Crystal size (mm)	0.02 × 0.02 × 0.01
Crystal system	Monoclinic
Space group	$C2/m$
a (Å)	10.742(2)
b (Å)	21.019(3)
c (Å)	11.787(2)
β (°)	117.06(3)
V (Å^3)	2370.0(7)
Z	2
**Data Collection**	
Instrument	Bruker APEX Kappa DUO (CCD) diffractometer
Radiation	Mo$K\alpha$
Average temperature (K)	293
2Θ range (°)	3.88 – 50.00
Total collected reflections	17382
Unique reflections	2141
Unique observed $\|F_o\|\geq 4\sigma_F$	1377
$R_{int.}$	0.106
hkl range	$-12<h<12$; $-24<k<24$; $-14<l<13$
**Refinement**	
Reflection file type	HKLF4
R_1 ($\|F_o\|\geq 4\sigma_F$)	0.028
R_1 (all data)	0.067
wR_2	0.054
S=$GooF$	0.955

Table S2. Crystal data, data collection and structure refinement details for lehmannite.

**Crystal Data**	
Formula	$(Na_{17.62}K_{0.38})_{\Sigma 18.00}(Ti^{4+}_{0.92}Sn_{0.08})_{\Sigma 1.00}Cu^{2+}_{12}(AsO_4)_8O_8Cl_{4.61}F_{1.18}$
Crystal size (mm)	0.01 × 0.01 × 0.01
Crystal system	Monoclinic
Space group	*C*2/*m*
a (Å)	10.8236 (15)
b (Å)	21.1077 (17)
c (Å)	11.8561 (11)
β (°)	117.195 (8)
V (Å^3)	2409.2 (5)
Z	2
**Data Collection**	
Instrument	Bruker APEX Kappa DUO (CCD) diffractometer
Radiation	Cu*K*α
Average temperature (*K*)	293
2Θ range (°)	8 – 135
Total collected reflections	7429
Unique reflections	2146
Unique observed $\|F_o\|\geq 4\sigma_F$	1531
$R_{int.}$	0.069
hkl range	$-12<h<12$; $-24<k<24$; $-14<l<13$
**Refinement**	
Reflection file type	HKLF4
R_1 ($\|F_o\|\geq 4\sigma_F$)	0.046
R_1 (all data)	0.072
wR_2	0.132
S=GooF	1.030

Table S3. Fractional atomic coordinates, site occupancies, multiplicities (*Q*) and isotropic displacement parameters (Å^2) of arsmirandite.

Site	Occupancy	*Q*	*x/a*	*y/b*	*z/c*	U_{iso}
A	$Na_{0.77}K_{0.23}$	4	1/2	0.74942(16)	1/2	0.0191(12)
Na2	$Na_{1.00}$	8	0.3665(3)	0.74992(13)	-0.2661(2)	0.0204(6)
Na3	$Na_{1.00}$	4	1/2	0.6397(2)	0	0.0203(8)
Na4	$Na_{1.00}$	8	0.2504(2)	0.63302(12)	-0.4998(2)	0.0203(6)
Na5	$Na_{1.00}$	4	1/4	3/4	0	0.0219(9)
Na6	$Na_{1.00}$	4	0.6390(4)	1/2	0.2787(4)	0.0228(9)
Na7	$Na_{1.00}$	2	0	1/2	1/2	0.0196(13)
Na8	$Na_{1.00}$	2	1/2	1/2	1/2	0.0202(13)
M	$Fe_{0.78}Mg_{0.11}Ti_{0.11}$	2	0	1/2	0	0.0123(7)
Cu1	$Cu_{1.00}$	4	0.32108(11)	1/2	0.21556(10)	0.0145(3)
Cu2	$Cu_{1.00}$	4	0.10550(11)	1/2	-0.21571(10)	0.0145(3)
Cu3	$Cu_{1.00}$	8	0.21337(8)	0.60787(4)	0.00003(7)	0.01416(19)
Cu4	$Cu_{1.00}$	8	0.10678(8)	0.60686(4)	0.21366(7)	0.01261(18)
As1	$As_{1.00}$	8	0.41317(6)	0.63996(3)	0.27995(5)	0.00978(15)
As2	$As_{1.00}$	8	0.13323(6)	0.64006(3)	-0.27998(5)	0.00999(15)
Cl1	$Cl_{1.00}$	2	1/2	1/2	0	0.108(3)
Cl2	$Cl_{1.00}$	4	0.7839(2)	1/2	0.5690(2)	0.0207(5)
Cl3	$Cl_{1.00}$	4	1/2	0.78445(11)	0	0.0186(5)
O1	$O_{1.00}$	8	0.2436(4)	0.65607(19)	-0.1262(3)	0.0149(10)
O2	$O_{1.00}$	8	0.3704(4)	0.6560(2)	0.1273(3)	0.0155(10)
O3	$O_{1.00}$	8	0.4623(4)	0.56348(19)	0.3122(3)	0.0148(10)
O4	$O_{1.00}$	8	0.1496(5)	0.56374(19)	-0.3122(3)	0.0163(10)
O5	$O_{1.00}$	8	0.2776(4)	0.65524(19)	0.3096(4)	0.0141(10)
O6	$O_{1.00}$	8	0.0331(4)	0.3451(2)	0.3106(4)	0.0173(10)
O7	$O_{1.00}$	8	0.5466(4)	0.6855(2)	0.3711(4)	0.0152(10)
O8	$O_{1.00}$	8	0.0609(4)	0.5598(2)	-0.1201(3)	0.0145(10)
O9	$O_{1.00}$	8	0.1757(4)	0.6854(2)	-0.3702(4)	0.0154(10)
O10	$O_{1.00}$	8	0.8193(4)	0.44017(19)	0.8790(3)	0.0123(9)

Table S4. Anisotropic displacement parameters of arsmirandite.

Site	U_{11}	U_{22}	U_{33}	U_{23}	U_{13}	U_{12}
A	0.020(2)	0.017(2)	0.0165(17)	0	0.0047(14)	0
Na2	0.0186(16)	0.0144(14)	0.0289(14)	-0.0012(12)	0.0113(12)	-0.0051(13)
Na3	0.0100(17)	0.033(2)	0.0210(19)	0	0.0097(15)	0
Na4	0.0186(15)	0.0238(16)	0.0146(11)	-0.0038(13)	0.0042(10)	-0.0068(13)
Na5	0.031(2)	0.012(2)	0.0218(19)	0.0015(17)	0.0113(18)	0.0028(17)
Na6	0.0127(18)	0.022(2)	0.039(2)	0	0.0165(16)	0
Na7	0.017(3)	0.026(4)	0.010(2)	0	0.000(2)	0
Na8	0.020(3)	0.029(4)	0.010(2)	0	0.005(2)	0
M	0.0130(12)	0.0115(13)	0.0093(11)	0	0.0024(8)	0
Cu1	0.0193(7)	0.0071(7)	0.0155(6)	0	0.0066(5)	0
Cu2	0.0176(7)	0.0084(7)	0.0158(6)	0	0.0061(5)	0
Cu3	0.0174(4)	0.0146(4)	0.0077(3)	-0.0002(4)	0.0033(3)	-0.0001(4)
Cu4	0.0123(4)	0.0135(4)	0.0125(4)	-0.0015(3)	0.0060(3)	-0.0028(3)
As1	0.0101(3)	0.0087(4)	0.0082(3)	0.0000(3)	0.0022(2)	-0.0004(3)
As2	0.0126(3)	0.0075(4)	0.0087(3)	-0.0008(3)	0.0038(3)	-0.0006(3)
Cl1	0.210(8)	0.042(4)	0.031(3)	0	0.018(4)	0
Cl2	0.0202(13)	0.0141(13)	0.0293(13)	0	0.0126(11)	0
Cl3	0.0147(12)	0.0221(15)	0.0176(11)	0	0.0063(9)	0
O1	0.018(2)	0.014(3)	0.007(2)	-0.007(2)	0.0014(18)	-0.0001(18)
O2	0.016(2)	0.015(3)	0.009(2)	-0.005(2)	-0.0003(18)	-0.0010(18)
O3	0.024(3)	0.007(2)	0.009(2)	0.002(2)	0.0036(18)	0.0019(18)
O4	0.028(3)	0.007(2)	0.012(2)	-0.001(2)	0.0077(19)	-0.0039(18)
O5	0.015(2)	0.012(3)	0.013(2)	0.0015(19)	0.0049(17)	-0.0022(18)
O6	0.020(2)	0.021(3)	0.008(2)	0.001(2)	0.0040(18)	0.0017(18)
O7	0.010(2)	0.014(3)	0.018(2)	-0.008(2)	0.0035(18)	-0.0055(19)
O8	0.016(2)	0.014(3)	0.007(2)	0.001(2)	-0.0001(17)	-0.0013(18)
O9	0.020(3)	0.013(3)	0.013(2)	-0.001(2)	0.0070(19)	0.0000(18)
O10	0.017(2)	0.013(3)	0.012(2)	0.0098(19)	0.0101(18)	0.0072(18)

Table S5. Fractional atomic coordinates, site occupancies, multiplicities (*Q*) and isotropic displacement parameters (Å^2) of lehmannite.

Site	*Q*	*x/a*	*y/b*	*z/c*	U_{iso}
*A**	4	1/2	0.7499(2)	1/2	0.0271(18)
Na2	8	0.8668(4)	0.75026(16)	0.7341(4)	0.0292(8)
Na3	4	1/2	0.6376(3)	0	0.0320(11)
Na4	8	0.2499(4)	0.63304(18)	0.5002(3)	0.0287(8)
Na5	4	3/4	3/4	0	0.0271(11)
Na6	4	0.6375(6)	1/2	0.2750(6)	0.0345(12)
Na7	2	0	1/2	1/2	0.0262(15)
Na8	2	1/2	1/2	1/2	0.0284(16)
*M***	2	0	1/2	0	0.0203(10)
Cu1	4	0.31871(19)	1/2	0.21457(16)	0.0208(4)
Cu2	4	0.89554(19)	1/2	0.21429(16)	0.0208(4)
Cu3	8	0.78880(13)	0.60721(6)	0.00016(11)	0.0209(3)
Cu4	8	0.10675(13)	0.60703(6)	0.21374(11)	0.0204(3)
As1	8	0.41227(9)	0.64007(4)	0.28016(8)	0.0175(3)
As2	8	0.36792(9)	0.85987(4)	0.28022(8)	0.0174(3)
O1	8	0.7586(7)	0.6568(3)	0.1259(5)	0.0233(13)
O2	8	0.3677(7)	0.6566(3)	0.1257(5)	0.0241(13)
O3	8	0.4608(7)	0.5634(3)	0.3121(6)	0.0250(13)
O4	8	0.8514(7)	0.5635(3)	0.3126(5)	0.0227(13)
O5	8	0.2771(6)	0.6556(3)	0.3108(5)	0.0212(12)
O6	8	1.0337(7)	0.6553(3)	0.3109(6)	0.0231(13)
O7	8	0.5466(6)	0.6856(3)	0.3710(5)	0.0192(12)
O8	8	0.9429(6)	0.5589(3)	0.1188(6)	0.0224(13)
O9	8	0.3251(6)	0.8143(3)	0.3712(5)	0.0211(13)
O10	8	0.8243(7)	0.5601(3)	-0.1196(6)	0.0256(14)
Cl1***	4	0.526(4)	1/2	-0.002(5)	0.051(9)
F****	4	0.6461(15)	1/2	0.0027(13)	0.025(3)
Cl2	4	0.7846(3)	1/2	0.5687(3)	0.0279(7)
Cl3	4	1/2	0.78429(15)	0	0.0282(7)

*Refined as ($Na_{0.81}K_{0.19}$); **refined as ($Ti_{0.92}Sn_{0.08}$); ***site occupancy ($Cl_{0.31}\square_{0.69}$); site occupancy ($F_{0.39}O_{0.30}\square_{0.31}$).

Table S6. Anisotropic displacement parameters of lehmannite.

Site	U_{11}	U_{22}	U_{33}	U_{12}	U_{13}	U_{23}
A	0.036(3)	0.023(3)	0.019(3)	0	0.010(2)	0
Na2	0.036(3)	0.023(3)	0.019(3)	0	0.010(2)	0
Na3	0.032(2)	0.0250(18)	0.0284(18)	0.0042(15)	0.0121(16)	0.0062(15)
Na4	0.026(3)	0.042(3)	0.028(3)	0	0.012(2)	0
Na5	0.0301(19)	0.0326(19)	0.0211(17)	-0.0040(15)	0.0095(15)	-0.0077(15)
Na6	0.036(3)	0.018(2)	0.028(3)	0.000(2)	0.015(2)	0.0005(19)
Na7	0.029(3)	0.034(3)	0.043(3)	0	0.019(3)	0
Na8	0.036(4)	0.026(4)	0.009(3)	0	0.004(3)	0
M	0.0256(17)	0.0179(16)	0.0144(16)	0	0.0065(12)	0
Cu1	0.0272(10)	0.0133(8)	0.0158(9)	0	0.0045(8)	0
Cu2	0.0309(10)	0.0130(8)	0.0165(9)	0	0.0089(8)	0
Cu3	0.0278(7)	0.0191(6)	0.0103(6)	0.0026(5)	0.0039(5)	0.0003(5)
Cu4	0.0262(7)	0.0181(6)	0.0142(6)	-0.0024(5)	0.0069(5)	-0.0041(5)
As1	0.0222(5)	0.0145(5)	0.0107(4)	-0.0011(3)	0.0030(4)	-0.0001(3)
As2	0.0244(5)	0.0142(5)	0.0105(4)	-0.0013(3)	0.0054(4)	0.0003(3)
O1	0.033(4)	0.021(3)	0.010(3)	0.008(2)	0.005(3)	0.006(2)
O2	0.032(3)	0.024(3)	0.013(3)	-0.004(3)	0.007(3)	0.001(2)
O3	0.031(3)	0.014(3)	0.019(3)	-0.004(2)	0.002(3)	-0.001(2)
O4	0.037(4)	0.012(3)	0.020(3)	0.000(2)	0.014(3)	0.000(2)
O5	0.023(3)	0.019(3)	0.018(3)	0.000(2)	0.007(2)	-0.007(2)
O6	0.028(3)	0.021(3)	0.016(3)	-0.003(2)	0.007(3)	-0.002(2)
O7	0.022(3)	0.015(3)	0.015(3)	-0.007(2)	0.003(2)	-0.001(2)
O8	0.027(3)	0.014(3)	0.021(3)	0.002(2)	0.008(3)	0.004(2)
O9	0.029(3)	0.019(3)	0.015(3)	-0.006(2)	0.009(3)	-0.003(2)
O10	0.037(4)	0.015(3)	0.020(3)	0.002(2)	0.008(3)	0.001(2)
Cl1	0.08(3)	0.028(4)	0.043(6)	0	0.023(16)	0
F	0.020(8)	0.028(7)	0.021(7)	0	0.006(6)	0
Cl2	0.0308(17)	0.0245(14)	0.0264(16)	0	0.0113(13)	0
Cl3	0.0285(16)	0.0312(16)	0.0218(14)	0	0.0088(13)	0

Table S7. Interatomic bond lengths (Å) in arsmirandite and lehmannite.

**Bond**	**Arsmirandite**	**Lehmannite**	**Notes**
A–O6	2 × 3.143(8)	2 × 3.148(8)	Octahedron [AO_6]
A–O7	2 × 2.248(4)	2 × 2.268(6)	(*A* = Na, K)
A–O9	2 × 2.269(4)	2 × 2.263(7)	
Na2–Cl3	2.886(3)	2.898(4)	Square pyramid [NaO_4Cl]
Na2–O5	2.431(5)	2.438(7)	
Na2–O6	2.439(9)	2.446(7)	
Na2–O7	2.299(5)	2.309(7)	
Na2–O9	2.293(5)	2.308(7)	
Na3–O1	2 × 2.488(4)	2 × 2.531(7)	Octahedron [NaO_4Cl_2]
Na3–O2	2 × 2.491(4)	2 × 2.526(6)	(arsmirandite)
Na3–Cl1	2.937(4)	2.920(6) (Na3–F)	Octahedron [NaO_4FCl]
Na3–Cl3	3.042(5)	3.096(6)	(lehmannite)
Na4–O5	2.440(5)	2.442(7)	Square pyramid [NaO_4Cl]
Na4–O6	2.431(4)	2.436(7)	
Na4–O7	2.295(5)	2.311(7)	
Na4–O9	2.303(5)	2.313(7)	
Na4–Cl2	2.889(3)	2.901(4)	
Na5–O1	2 × 2.454(4)	2 × 2.446(6)	Octahedron [NaO_4Cl_2]
Na5–O2	2 × 2.462(4)	2 × 2.449(6)	
Na5–Cl3	2 × 2.7814(8)	2 × 2.8010(9)	
Na6–O3	2 × 2.493(5)	2 × 2.533(8)	Octahedron [NaO_4Cl_2]
Na6–O4	2 × 2.509(5)	2 × 2.531(8)	
Na6–Cl1	2.926(4)	2.92(6)	
Na6–Cl2	3.047(4)	3.097(7)	
Na7–O4	4 × 2.458(4)	4 × 2.460(6)	Octahedron [NaO_4Cl_2]
Na7–Cl2	2 × 2.787(2)	2 × 2.799(3)	
Na8–O3	4 × 2.455(4)	4 × 2.464(6)	Octahedron [NaO_4Cl_2]
Na8–Cl2	2 × 2.776(2)	2 × 2.803(3)	
M–O8	4 × 2.203(4)	4 × 2.170(6)	Cube [MO_8]
M–O10	4 × 2.206(4)	4 × 2.184(6)	

Table S7 (continued). Interatomic bond lengths (Å) in arsmirandite and lehmannite.

**Bond**	**Arsmirandite**	**Lehmannite**	**Notes**
Cu1–O3	2 × 1.951(4)	2 × 1.967(6)	Square [CuO_4]
Cu1–O10	2 × 1.889(4)	2 × 1.918(6)	
Cu2–O4	2 × 1.950(4)	2 × 1.972(6)	Square [CuO_4]
Cu2–O8	2 × 1.889(4)	2 × 1.904(6)	
Cu3–O1	1.945(4)	1.967(6)	Square [CuO_4]
Cu3–O2	1.955(4)	1.968(6)	
Cu3–O8	1.897(4)	1.912(6)	
Cu3–O10	1.905(4)	1.911(6)	
Cu4–O5	1.947(4)	1.959(6)	Square [CuO_4]
Cu4–O6	1.943(4)	1.956(6)	
Cu4–O8	1.906(4)	1.902(6)	
Cu4–O10	1.895(4)	1.883(6)	
As1–O2	1.676(4)	1.703(6)	Tetrahedron [AsO_4]
As1–O3	1.681(4)	1.689(6)	
As1–O5	1.676(4)	1.693(6)	
As1–O7	1.651(4)	1.662(5)	
As2–O1	1.688(4)	1.701(6)	Tetrahedron [AsO_4]
As2–O4	1.676(4)	1.692(6)	
As2–O6	1.684(4)	1.691(6)	
As2–O9	1.640(4)	1.662(6)	

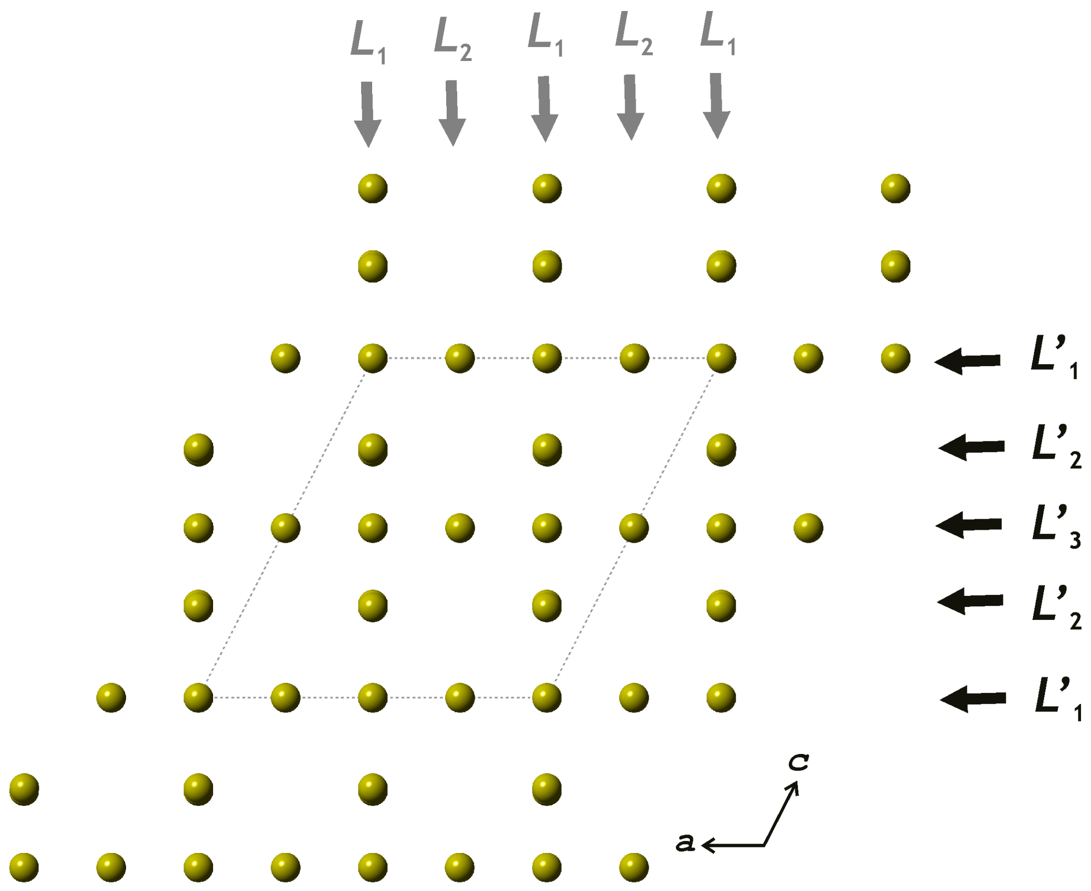

Figure S1. The Na cation array in the crystal structure of lehmannite and its splitting into layers shown in Figure S2.

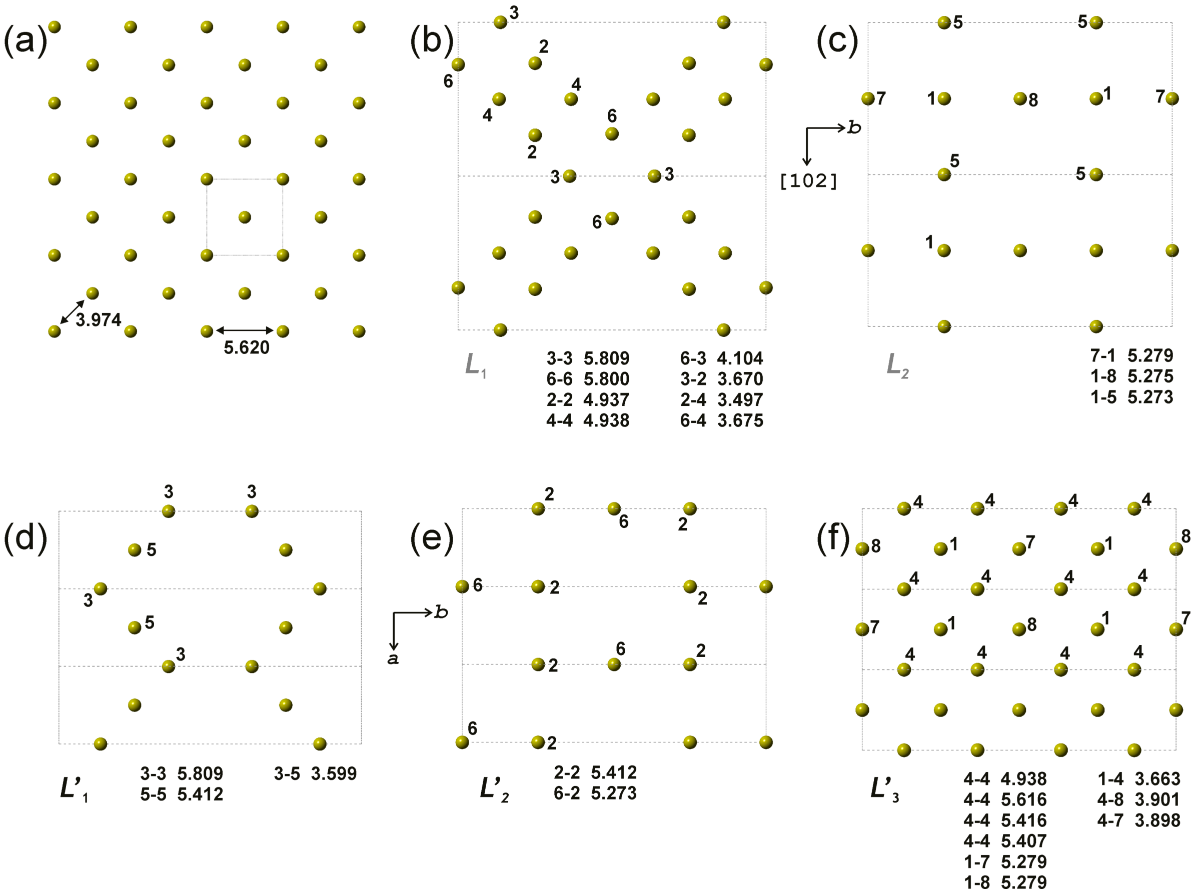

Figure S2. The (100) section of the Na array in halite, NaCl (a) and the layers of Na atoms in the crystal structure of lehmannite (b-f; see notations of the layers in Figure S1). The numbering of the Na atoms correspond to the crystal-structure data. The principal Na···Na interatomic distances (in Å) are given below the layers.

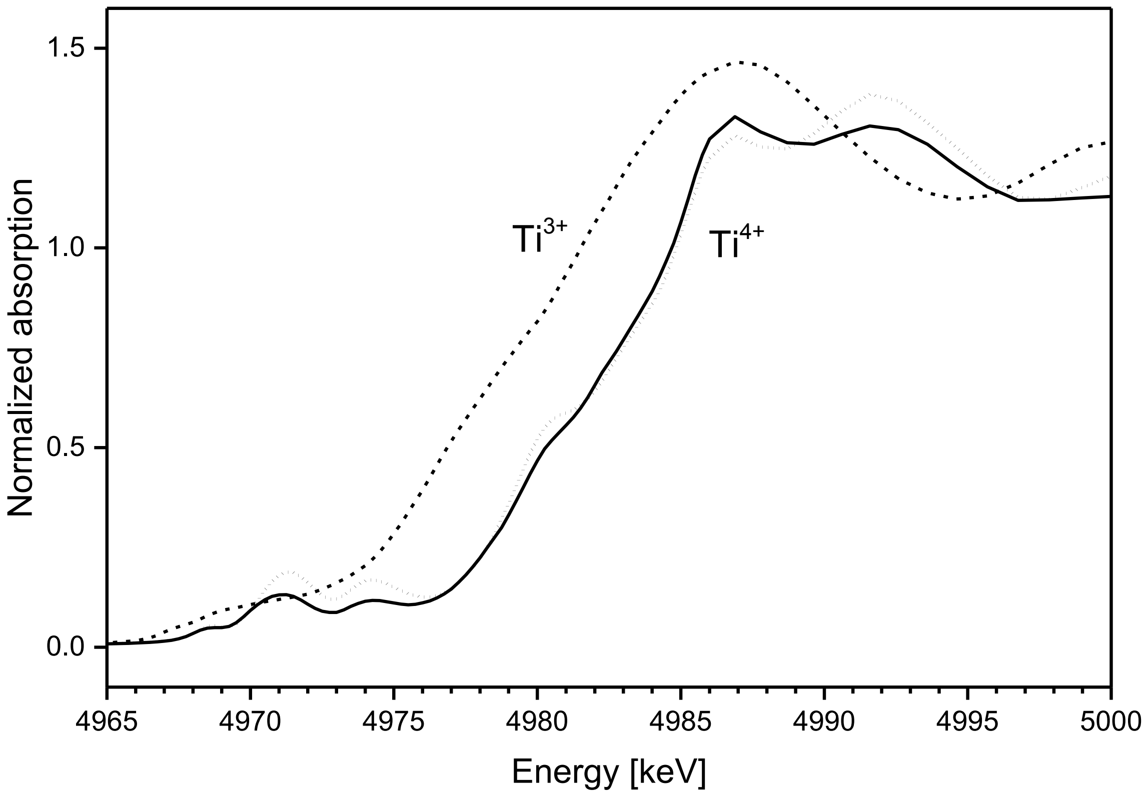

Figure S3. The Ti *K* XANES spectrum of lehmannite (solid line) compared to the spectra of Ti_2O_3 as reference for Ti(III) (dashed line) and of rutile TiO_2 as reference for Ti(IV) (dotted line).

Lehmannite was studied at the SUL-X beamline of the ANKA synchrotron ring (Karlsruhe Institute of Technology). Ti*K*α fluorescence emission intensities were recorded with a 7 element Si(Li) solid state detector (RAYSPEC) and divided by the incoming signal intensity using an ADC ionization chamber. Spectra were merged and further processed using the Athena program of the IFFEFIT software package[2]. Ti *K* XANES spectra of Ti_2O_3 as reference for Ti(III) and two modifications of TiO_2 (rutile and brookite), $FeTiO_3$ (ilmenite) and $CaTiOSiO_4$ (titanite) as references for Ti(IV) were measured in transmission using ionization chambers form company ADC. The match with the Ti(IV) edge position is shown in Figure S3 in comparison with the rutile reference spectrum.

References

1. Sheldrick, G. M. Crystal structure refinement with SHELXL. *Acta Crystallogr.* **C71**, 3-8 (2015).
2. Ravel, B. & Newville, M. ATHENA, ARTEMIS, HEPHAESTUS: Data analysis for X-ray absorption spectroscopy using IFEFFIT. *J. Synchrotron Rad.* **12**, 537-541 (2005).
